# COVID-19 vaccines for patients with cancer: benefits likely outweigh risks

**DOI:** 10.1186/s13045-021-01046-w

**Published:** 2021-02-27

**Authors:** Joyce K. Hwang, Tian Zhang, Andrew Z. Wang, Zihai Li

**Affiliations:** 1 Department of Medicine, Durham, NC USA; 2 Division of Medical Oncology, Department of Medicine, Duke Cancer Institute, DUMC Box 103861, Durham, NC 27710 USA; 3 Duke Cancer Institute Center for Prostate and Urologic Cancers, Durham, NC USA; 4 Department of Radiation Oncology, University of North Carolina Chapel Hill, Chapel Hill, NC USA; 5 Pelotonia Institute for Immuno-Oncology, The OH State University Comprehensive Cancer Center – James, Columbus, OH USA

**Keywords:** COVID-19, COVID-19 vaccines, SARS-CoV-2 virus vaccines, COVID-19 and cancer, Vaccination, Cancer therapies, Patients with cancer and COVID-19

## Abstract

Less than a year since the start of the COVID-19 pandemic, ten vaccines against SARS-CoV-2 have been approved for at least limited use, with over sixty others in clinical trials. This swift achievement has generated excitement and arrives at a time of great need, as the number of COVID-19 cases worldwide continues to rapidly increase. Two vaccines are currently approved for full use, both built on mRNA and lipid nanotechnology platforms, a success story of mRNA technology 20 years in the making. For patients with cancer, questions arise around the safety and efficacy of these vaccines in the setting of immune alterations engendered by their malignancy and/or therapies. We summarize the current data on leading COVID-19 vaccine candidates and vaccination of patients undergoing immunomodulatory cancer treatments. Most current cancer therapeutics should not prevent the generation of protective immunity. We call for more research in this area and recommend that the majority of patients with cancer receive COVID vaccinations when possible.

## Natural immunity to SARS-CoV-2

**Fig. 1:**
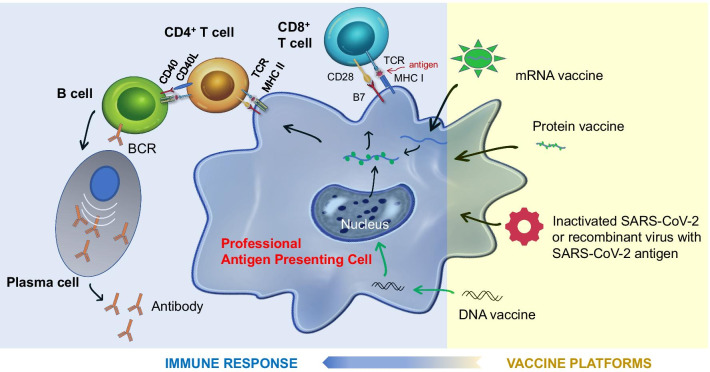
Different types of COVID19 vaccines in development, mechanisms of antigen presentation, and generation of protective immunity

Protective immunity against viral infections involves humoral immunity and cell-mediated immunity (Fig. 1). Humoral immunity is provided by B lymphocytes which produce antibodies which may neutralize virus by binding virus and preventing its entry into host cells. Cell-mediated immunity includes macrophages and CD8^+^ cytotoxic T lymphocytes, which eliminate infected cells. CD4^+^ T lymphocytes help to activate B and CD8^+^ T cells, which promote the generation of highly effective antibody responses and memory. CD4^+^ helper T cell subsets include Th1 which promotes cell-mediated immunity and opsonizing IgG antibodies, and Th2 which promotes IgE antibodies and, broadly, allergic-type inflammation. Following infection, antigen-specific memory B and T cells persist and recall immune responses upon repeat encounter. In a viral infection, these protective immune responses are initiated by professional antigen-presenting cells such as dendritic cells, which capture, process, and display viral peptides to MHC molecules to prime naïve antigen-specific T cells in the secondary lymphoid tissues. Productive T cell priming often requires additional stimulatory cytokines and co-stimulatory molecules. The goal of a vaccine is to provide stimulation by the desired antigen(s) in a context that mimics infection enough to elicit protective memory immunity with a tolerable safety profile. Productive immunogenic vaccines often require adjuvants and/or a "prime-boost" strategy of repeated doses to enhance durable immune responses.

What immune responses are desired for protective immunity against SARS-CoV-2? Re-challenge models in rhesus macaques indicated that primary exposure to SARS-CoV-2 is protective against re-infection [1]. Convalescent patients after COVID-19 infections have high levels of neutralizing antibodies, particularly to the spike glycoprotein of the virus, which mediates host cell attachment and viral entry [2]. Animal models indicate that passive transfer of neutralizing antibodies are protective against SARS-CoV-2 infection [3, 4], and early administration of convalescent plasma protects against progression of COVID-19 disease in human trials[5]. However, antibodies are not sufficient, at least for clearing established disease, since high titers of neutralizing antibodies are found in patients with severe disease [6]. In this regard, convalescent COVID-19 patients also have high levels of virus-specific T cells [7]. CD4^+^ T-cell responses appear to be at least as important as CD8^+^ T-cell responses and recognize the spike protein among others [8, 9]. Neutralizing antibody, memory B and memory T cells specific to SARS-CoV-2 have now been found in convalescent patients after six months [2, 10, 11]. In addition, one study identified a subset of individuals who rapidly resolved symptoms after being infected by COVID-19 and found that they sustained both antibody production and memory CD4^+^ T-cells several months after infection [9]. While immune correlates of protection have yet to be defined in humans, the data overall support that the integration of humoral and cell-mediated immunity to SARS-CoV-2 are the key to protection and can be highly effective and durable.

There are also immune responses that would be undesirable from a vaccine. In studies of SARS-CoV-1, immunization with inactivated virus provoked Th2-mediated lung immunopathology upon infection [12, 13]. This immunopathology was observed in animal data and has not been observed for SARS-CoV-2 in non-human primate models [14, 15]. Nevertheless, investigators seek to minimize this risk by eliciting a Th1-skewed CD4^+^ T-cell response. Another concern that has been raised in the literature is that of antibody-dependent enhancement (ADE) of disease, mediated by antibodies that bind to but do not neutralize the virus. ADE takes two main forms [16]. In one form, well characterized in dengue infections, virus is bound by antibody and the antibody-virus complex is internalized via interactions with Fc gamma receptor into macrophages where it replicates. In the other form, non-neutralizing antibodies mediate the formation of immune complexes, which incite inflammation. ADE has been proposed as a concern for COVID-19 vaccine design due to the finding of high levels of antibodies in severe COVID-19 and in vitro observations that SARS-CoV-2 is taken up by macrophages [16]. However, there are explanations for high antibody levels in severe diseases other than antibody-mediated harm, including chronic antigen stimulation and insufficiency of the antibody response to clear established disease as described above. Importantly, SARS-CoV-2 does not productively replicate in macrophages, making the macrophage-mediated type of ADE less relevant. There is no compelling evidence of ADE from convalescent plasma trials [5, 17, 18] or any of the human vaccine trials thus far. Nevertheless, the likelihood of ADE is theoretically diminished by vaccines that focus the elicited antibody response on neutralizing epitopes.

## SARS-CoV-2 vaccines in use:

At the time of writing, an astounding 66 COVID-19 vaccines are being tested in clinical trials, with 10 approved for at least limited use. Multiple platforms are represented among the leading candidates [19, 20], each with pros and cons (Table 1).

**Table 1:** Advantages and disadvantages of various types of COVID19 vaccines in development

Vaccine type	COVID-19 vaccines furthest in development/approved	Advantages	Disadvantages
Inactivated virus	SinoVac (CoronaVac + aluminum) SinoPharm (Inactivated whole virus SARS-CoV-2 + aluminum)	Entire virus, with all antigens presented Prior experience and technology – e.g., quadrivalent influenza vaccine Easier storage – does not need to be frozen	Need adjuvants to boost Poor inducers of CD8 + T-cell immunity Hard to mass-produce Large batches of live virus pose biosecurity risk
Protein subunits	Novavax (NVX-CoV2373) Vector Institute (EpiVacCorona)	Can focus on antigens that generate neutralizing antibodies Does not introduce intact pathogen	Produced ex vivo, may not retain post-translational modifications or conformation Not efficiently presented Lower humoral and cellular response Require adjuvants to boost
Replication incompetent adenoviral vector	AstraZeneca (ChAdOx1 nCoV-19; AZD1222) Johnson & Johnson (Ad26.COV2.S) CanSino Biologics (Ad5-nCoV) Gamaleya (Sputnik V)	Replication-defective, no new viral particles Avoids intact pathogen Mimics natural infection Elicits humoral and cellular immunity	Anti-vector immunity may interfere Lower efficacy if prior anti-vector immunity exists
DNA	Inovio (INO-4800)	Mimic natural infection Elicits strong humoral and cellular immunity Avoids introducing pathogen Easier to mass-produce	Delivery into cell nucleus
mRNA	Moderna (mRNA-1273) Pfizer-BioNTech (BNT162b2)	Delivery into cytoplasm Unable to integrate into host genome Elicit strong humoral and cellular immunity Avoids anti-vector immunity Avoids introducing pathogen Easier to mass-produce	Fragile – easily degraded Needs lipid nanoparticle for delivery Frozen for storage

### Inactivated vaccines

Inactivated vaccines contain intact microbe that has been killed (e.g. the quadrivalent influenza vaccine). Inactivated vaccines present the entire virus and thus breadth of antigens to the immune system, but may require an adjuvant to boost immunogenicity and are generally poor inducers of CD8^+^ T cell immunity. Moreover, production of these vaccines is slow because it requires large-scale culture and inactivation. CoronaVac, a chemically inactivated vaccine by **SinoVac**, is provided in two doses two weeks apart and uses aluminum adjuvant. In phase I/II trials, it produced no serious adverse events and > 90% seroconversion, but lower titers than in convalescent patients, and low T cell responses [21]. CoronaVac is licensed for limited use in China while phase III trials are underway. **Sinopharm** has developed two other inactivated vaccines which induced humoral responses in humans in Phase I/II studies [22] and are in limited use. Of note, aluminum adjuvants have been noted to initiate Th2 responses. In addition, ADE is theoretically more likely with inactivated intact vaccines due to the inclusion of non-neutralizing epitopes. Nevertheless, there have been no reports of ADE.

### Protein subunit vaccines

Subunit vaccines contain an isolated protein of the target pathogen, produced recombinantly in cell culture and purified (e.g., hepatitis B vaccine). Subunit vaccines circumvent dangers associated with introducing intact pathogen and the specific protein can be chosen to focus the immune response on particular epitopes (e.g., neutralizing epitopes). Subunit vaccines stimulate CD4^+^ T-cell and antibody responses. However, since the protein is produced ex vivo in cell culture, it may not retain native post-translational modifications or conformation. In addition, exogenous isolated proteins are not efficiently presented via the pathways that stimulate cytotoxic T cells, so subunit vaccines generally require adjuvants for immunogenicity. NVX-CoV2373, developed by **Novavax,** contains full length spike protein produced from insect cells. In phase I/II trials [23], NVX-CoV2373 elicited high neutralizating antibody titers when delivered with adjuvant, exceeding titers in a panel of convalescent patients. Th1-type CD4^+^ T-cell responses were observed in most participants. CD8^+^ T-cell responses were not reported. No serious adverse events were noted, and reactogenicity was generally mild to moderate, with fever in only 1 of 83 vaccinated participants. Phase III trials are underway.

### Replication-incompetent viral vectored vaccines

Viral-vectored vaccines contain a delivery virus (e.g., adenovirus) that has been recombinantly engineered to contain genes encoding antigens of choice from the target pathogen. Upon inoculation, the engineered virus infects host cells, leading to expression of the vaccine antigen. Often the recombinant virus is rendered replication-defective, so that host cells can be infected but cannot form new viral particles. Viral vector vaccines avoid introduction of intact target pathogen and result in endogenous antigen production mimicking natural infection, and thus are expected to elicit both humoral and cellular immunity. A disadvantage is that anti-vector immunity may interfere with prime-boost strategies or lead to low efficacy from preexisting anti-vector immunity.

ChAdOx1 nCoV-19 (also known as AZD1222), developed by **AstraZeneca**, is a replication-deficient chimpanzee recombinant adenoviral vector vaccine containing the SARS-CoV-2 spike protein administered as a two-dose prime-boost regimen. The interim analysis of randomized controlled trials of ChAdOx1 nCoV-19 found a vaccine efficacy of 62%-90% depending on dosing regimen and age [24, 25]. Of 10 participants hospitalized due to COVID-19 including one fatal case, all were in the control group. Reactogenicity was more common with the vaccine versus control. Serious adverse events were overall balanced between experimental and control groups, but did include a case of transverse myelitis following ChAdOx1 nCoV-19 booster vaccination. This case led to a temporary pause in the phase II/III trial. The vaccine was eventually authorized for emergency use in the UK, Argentina, and India. Unlike the approved mRNA vaccines (see below), it is stable for months at refrigeration temperatures.

Ad5-nCoV, produced by **CanSino Biologics**, is a replication-deficient adenovirus type-5 vectored vaccine containing full-length spike gene administered as a single dose [26]. Phase I studies showed good tolerability and immunogenicity with induction of both specific T-cell and humoral responses. However, the phase II study [27] showed induction of S-protein neutralizing antibodies in only 47–59% of vaccinated participants, thought to be due to preexisting immunity to adenovirus type 5. This vaccine has been approved for limited use in China.

Ad26.COV2.S developed by **Johnson & Johnson** uses a non-replicating adenovirus-serotype 26 vector expressing full-length spike glycoprotein. A single dose elicited strong neutralizing antibodies and protection against SARS-CoV-2 challenge in rhesus macaques [28]. Phase I/IIa interim results [29] reported one vaccine-related serious adverse event (fever leading to hospitalization). There was a trend for overall higher reactogenicity with younger age, including the occurrence of fevers in 19% of younger participants and 4% of older participants. The seroconversion rate was 83–100% at day 29 by viral neutralization assays depending on dose and age of recipient. Cell-mediated responses were also observed: 80–83% of patients had Th1-skewed CD4^+^ T-cell responses, and 51–64% had CD8^+^ T cell responses. If approved, pending phase III trial results, Ad26.COV2.S would have the advantages of potentially being administered as a single-dose regimen and stable storage at refrigeration temperatures.

Other candidates include Sputnik V [30] developed by **Gamaleya**, which employs two different adenoviral vectors in a heterologous prime-boost strategy to circumvent anti-vector immunity to the prime. Phase I/II trials reported elicitation of humoral and cell-mediated responses in all participants, without serious adverse events. Sputnik V is in use in Russia with peer review of phase III trial results pending.

### Nucleic acid vaccines

Nucleic acid vaccines contain nucleic acids encoding a protein antigen. Upon inoculation, the nucleic acids are taken up by antigen presenting cells and expressed. Like viral-delivered nucleic acids (i.e., viral vectored vaccines), nucleic acid vaccines mimic natural infection with endogenous antigen production and eliciting strong T and B cell responses, while being entirely non-infectious. Making gene constructs at scale is more rapid than producing recombinant protein or inactivated pathogens, which is advantageous for vaccination against emerging virus variants which has already been reported for SARS-CoV-2 worldwide [31].

Nucleic acid vaccine candidates include DNA plasmid vaccines and mRNA vaccines. A spike-protein expressing DNA vaccine was shown to elicit humoral and cellular immunity in rhesus macaques [32]. Four DNA vaccines are in at least phase II or III trials including **Inovio's** INO-4800, with efficacy data in humans pending. mRNA vaccines have additional advantages over DNA and other vaccine platforms [33, 34]. mRNA is the normal intermediate between protein-encoding DNA and the production of protein in the cytoplasm. Thus whereas DNA vaccines need to be delivered into the cell nucleus, mRNA vaccines are delivered to the cytosol, avoiding risk of host genome integration. mRNA is the minimal genetic vector for translation of the antigen, avoiding anti-vector immunity. Furthermore, mRNA is transient, degraded by ubiquitous RNAses and normal cellular processes. While mRNA vaccines had not previously been licensed for human use, they have been under development since the 1990s, including as cancer vaccines encoding neoepitopes with early trials in patients with melanoma. [35, 36]. Advancements in mRNA delivery, particularly lipid nanoparticle (LNP) technology enabling the encapsulation and endosomal release of mRNA to the cytoplasm, have been key innovations that overcame initial concerns about the intrinsic fragility of mRNA and inefficient in vivo delivery [37]. Also key were discoveries of how to modulate the intrinsic immunogenicity of mRNA using modified nucleosides [38]. By 2017, Phase I trials had been performed of LNP-encapsulated mRNA vaccines against influenza, showing high seroconversion rates with an excellent safety profile [39].

Both of the vaccines approved under emergency use in the United States – BNT162b2, developed by **Pfizer and BioNTech**, and mRNA-1273, developed by **Moderna** – are LNP-formulated, nucleoside-modified mRNA vaccines encoding the SARS-CoV-2 full-length spike protein modified by two proline mutations to lock it in pre-fusion conformation, given as a two-dose prime-boost regimen 21 days (for BNT162b2) or 28 days (for mRNA-1273) apart.

Phase I studies of BNT162b2 showed 100% anti-spike seropositivity by day 21, boosted further by day 28 to titers above those of a COVID-19 human convalescent panel [40]. A follow-up preprint [41] reported also expansion of spike-specific CD8^+^ and Th1 subtype CD4^+^ T cell responses, with a high fraction producing interferon-γ. In the phase III trial, 43,548 participants were randomized to two doses of BNT162b2 versus placebo, 37,706 of whom had sufficient follow up (median 2 months) [42]. There were 162 cases of laboratory-confirmed symptomatic COVID-19 among 18,325 participants in the placebo cohort, compared to 8 cases among 18,198 participants in the vaccinated cohort, yielding a vaccine efficacy of 95%. Efficacy was maintained (> 91%) across age and underlying medical conditions. There were 10 cases of severe COVID-19, 9 in the placebo cohort and 1 in the vaccine cohort. Reactogenicity was frequent and mostly mild to moderate, with more than half of vaccinated participants experiencing fatigue and headaches, particularly after the second dose. Systemic adverse reactions resolved in a median of 1 day and included fever in 16% of younger participants and 11% of older participants [43]. Four patients out of 21,720 treated with at least one dose of vaccine developed Bell’s palsy at 3, 9, 37, and 48 days after vaccination and all resolved within 3 weeks (at 3, 10, 15, and 21 days, respectively). [43] 8.8% had grade ≥ 3 reactions (most common were fatigue, headache, muscle pain, chills, and injection site pain) [40]. Few had severe adverse events (1.1% in BNT162b2 cohort, 0.6% in the placebo cohort). Few had serious adverse events (0.6% vs 0.5%), of which 1.6% (shoulder injury, lymphadenopathy) were considered by the FDA to be likely related to the vaccine [43].

Phase I studies of mRNA-1273 showed 100% anti-spike seropositivity in all dose groups, and S-specific Th1 subtype CD4^+^ T-cell expansion. CD8^+^ T cell responses were detected at low levels. An updated analysis of the phase I study reports durability of the humoral response, with titers remaining above those of convalescent controls at 90 days after second immunization [44]. In the phase III trial, 30,420 participants were randomized to mRNA-1273 versus placebo. There were 185 cases of symptomatic COVID-19 infection in the placebo group and 11 in the vaccinated group, yielding a vaccine efficacy of 94.1% [45]. Efficacy was maintained (≥ 86%) across age, sex, race, and underlying medical conditions [46]. There were 30 cases of severe COVID-19, all in the placebo group [46]. Reactogenicity was frequent, mostly mild to moderate. Systemic adverse reactions resolved in a mean of 3 days. Grade ≥ 3 reactions occurred in 21.6% in the mRNA-1273 recipients versus 4.4% in placebo. Four Bell’s palsy cases were reported: 3 cases in 15,181 people treated in the vaccine group and 1 case in 15,170 people treated in the placebo group. These cases occurred at 22, 27, 28, and 32 days after vaccination, with resolution in 3 cases and one ongoing at the time of the FDA review. Few had serious adverse events (1.0% in vaccine recipients versus 1.0% in placebo), of which 2.1% (nausea/vomiting, facial swelling) were considered by the FDA to be likely related to vaccination [47].

In sum, both mRNA vaccines offer highly effective protection against symptomatic COVID-19 infection, mediated by a combined humoral and cell-mediated immune response with frequent reactogenicity but low to no serious adverse effects. mRNA-1273 has a logistical advantage over BNT162b2, being stable at refrigeration temperatures, compared to ultracold (-70 °C) temperatures for BNT162b2.

## Implications for patients with cancer

Patients with cancer are at increased risk of severe illness from COVID-19 [15, 48–51]. In a study of 73 million patients in the USA, of whom 273,000 had been diagnosed with cancer in the last year and 16,570 were diagnosed with COVID-19, patients with cancer had greatly increased odds of COVID-19 infection (adjusted odds ratio (aOR) of 7; [52]). Odds of infection were highest for patients with recently diagnosed leukemia (aOR 12.2), non-Hodgkin's lymphoma (aOR 8.5), and lung cancer (aOR 7.7). Mortality is also higher in patients with cancer who develop COVID-19: patients with cancer and COVID-19 have a greater risk of mortality (14.9%) than patients with COVID-19 without cancer (5.3%) and patients with cancer without COVID-19 (4.0%) [52]. For patients diagnosed with a hematologic malignancy in the last 5 years, the increased risk of death has been estimated to be at least 2.5-fold, and for other cancers, at least 1.2-fold [48].

Because of the increased vulnerability of patients with cancer to COVID-19 infections and mortality, there is urgent interest in vaccinating this population expeditiously. Considerations around expected safety and efficacy differ by therapy based on their general mechanisms and associated immune alterations.

### Considerations for patients treated with cytotoxic chemotherapies

Cytotoxic chemotherapies interfere with DNA replication, synthesis, and cell cycle progression. Lymphocytes proliferate rapidly as part of activation and so are suppressed by these therapies [53]. However, suppression is not complete and immune responses can nevertheless be elicited to vaccination while on cytotoxic chemotherapy. Patients with acute lymphoblastic leukemia, in which the immune system is directly impacted by disease as well as treatment, can still generate immune responses after vaccination, ranging from 10 and 27% of patients immunized with hepatitis B and meningococcal subunit vaccines, respectively, to 100% of patients immunized with diphtheria and tetanus toxoid vaccines [54–56]. In studies of responses to the annual inactivated influenza vaccine in patients with cancer, 10–42% of patients with hematologic malignancies responded to one dose of influenza vaccine [57–59], with additional responses with a second dose [57, 58]. Higher responses are seen in patients with solid tumors on chemotherapy [60]: at least 78% in patients with lung cancer [61] and 81% of patients with breast cancer [59] on mild to moderately immunosuppressive regimens. When given between cycles of chemotherapy for lung or breast cancer, timing relative to the last cycle may matter, though estimates of the optimal day varies [60, 62, 63]. Vaccination was well-tolerated in these studies. Infectious Diseases Society of America (IDSA) and The European Conference on Infections in Leukemia (ECIL) guidelines recommend yearly vaccination with inactivated influenza vaccine—an exception is during intensive therapy (e.g., induction and consolidation therapy for acute leukemias) given likely poor response, but considered reasonable given seasonal nature of influenza [64, 65]. Hepatitis B subunit and pneumococcus vaccinations may also be recommended even during chemotherapy [65, 66]. Titers can be helpful to assess need for revaccination [64, 66]. Higher doses or boosters are employed to enhance immunogenicity to inactivated influenza, pneumococcal polysaccharide, and hepatitis B subunit vaccines [64–66]. Overall, with the exception of during periods of intensive chemotherapy, patients undergoing chemotherapy are expected to generate protective responses with COVID-19 vaccination.

### Considerations for patients treated with targeted therapies

Targeted therapies include receptor tyrosine kinase inhibitors (TKIs) such as erlotinib, sunitinib, and imatinib or monoclonal antibodies such as trastuzumab. Targeted therapies should not directly cause immunosuppression as part of their mechanism of action, but may have unintended inhibitory effects on antigen presenting cell function, T cell activation [67] and B cell signaling [68]. Nevertheless, patients treated with sunitinib or sorafenib develop seroprotection with the influenza vaccine comparable to healthy controls [69]. Similarly, patients with chronic myelogenous leukemia (CML) on TKIs develop seroprotection after the influenza vaccine at reduced but still substantial rates around 40% [68]. There was also no difference in seroprotection against influenza when comparing controls versus patients with breast cancer treated with anti-HER2 monoclonal antibody trastuzumab [70]. Ibrutinib, an inhibitor of Bruton tyrosine kinase essential for B-cell receptor signaling, maturation, and immunoglobulin synthesis, unsurprisingly impairs responses, producing seroconversion in only 7–26% of patients after influenza vaccination [71, 72], though 75% of patients on ibrutinib were able to respond to subunit vaccines against varicella zoster [73]. The ECIL group recommends that patients with CML on TKIs receive the yearly inactivated influenza vaccine and to be vaccinated against *Streptococcus pneumoniae*. Thus, it is reasonable to expect that patients being treated with targeted therapies will generate protective responses with COVID-19 vaccination.

### Considerations for patients treated with immune checkpoint inhibitors

Immune checkpoint inhibitors target immunosuppressive pathways such as programmed cell death protein 1 (PD-1) and cytotoxic T-lymphocyte-associated protein 4 (CTLA-4) that are upregulated in tumor-reactive T cells, thereby enhancing immune responses and endogenous anti-tumor activity. While cancers such as lung cancers and comorbidities such as smoking have been associated with higher severity of COVID-19 infections [50, 74, 75], concurrent immune checkpoint inhibitor treatments for patients with lung cancer have not been associated with more severe infections or mortality when adjusted for smoking status [76].

Checkpoint inhibitors incur a risk of immune-related adverse events (IRAEs), at a rate of 17–48% for any grade and 5–8% severe grade, depending on the specific therapy [77]. There is a theoretical concern that vaccination could stimulate an overexuberant immune response and increase IRAEs in patients actively treated with immune checkpoint inhibitors. A 2018 study of 23 patients on immune checkpoint inhibitors who received the influenza vaccine found a high rate of IRAEs (52%). However, subsequent larger studies including three with non-vaccinated comparison groups did not show higher frequencies of IRAEs with vaccination [78, 79]. Moreover, immune checkpoint inhibitors are considered safe to use in patients with chronic HIV, hepatitis B, and hepatitis C infections, suggesting that stimulation by viral antigens is safe even in the context of bona fide infection [79]. Furthermore, influenza vaccine-induced seroprotection is generally not substantially diminished [78, 80]. Thus, we expect that patients on immune checkpoint inhibitor therapy should make protective responses with COVID-19 vaccination. Whether IRAEs increase after COVID-19 vaccinations warrants close study. In the interim, it may safe from a cancer treatment perspective to delay immune checkpoint inhibitor treatment in some settings [81].

### Considerations for patients treated with lymphodepleting or plasma cell depleting therapies

Lymphodepleting and plasma cell depleting therapies include anti-CD20 antibodies used for treatment of hematologic malignancies and autoimmune diseases as well as anti-CD38 monoclonal antibodies used in the treatment of multiple myeloma. Anti-CD20 treatments deplete peripheral B cells for at least 4 months [82, 83] and during this period impairs immune responses to vaccination including those against influenza, *Streptococcus pneumoniae*, and *Haemophilus influenza* [84, 85]. T cells may also be reduced as a consequence of the reduced pool of antigen-presenting B cells [84, 86].

Adoptive cellular immunotherapy targeting B cells to treat hematologic malignancies include CAR-T cells against CD19, which is expressed by nearly all B cells. Anti-CD19 therapy is B cell depleting, with high likelihood of subduing antibody responses to vaccination and increasing susceptibility to severe disease from COVID-19. There is little data on the immunogenicity and safety of vaccinations after CD19-targeted CAR-T cell therapy. Expert opinion of a committee of the National Comprehensive Cancer Network (NCCN) recommends that vaccination should be delayed for at least 3 months post-hematopoietic cell transplant or cellular therapy [87]. Previously established plasma cells may not be affected by anti-CD19 therapies owing to their lack of CD19 expression, so vaccine or pathogen-specific serum immunoglobulins may be maintained post-treatment [88].

Anti-CD38 therapies target plasma cells and are therefore also B-cell depleting. T cell activation may conversely be enhanced due to the expression of CD38 on immunosuppressive cell populations [89]. In patients with multiple myeloma treated with daratumumab, the frequency of normal plasma cells in bone marrow samples is decreased as well as levels of polyclonal immunoglobulins. However, IgG levels and induction of protective antibody titers were intact against *Streptococcus pneumoniae*, *Haemophilus influenzae B* and seasonal influenza at a median of 2 months after treatment, presumably due to a subset of plasma cells expressing reduced levels of CD38 that escape treatment [90].

In practice, it is recommended that vaccines be given at least 6 months after anti-B cell therapy due to likely futility [64, 66]. Despite expected reduced responses, an exception is made for the influenza vaccine which is given yearly, though ideally at least 2 weeks prior to lymphodepleting chemotherapies [91]. Patients on anti-B cell therapy are at especially high risk for severe disease and death from COVID-19 and prolonged viral shedding [92, 93], and thus, a similar exception would be reasonable to apply to COVID-19 vaccination.

### Considerations for patients treated with radiation

Radiation therapy is commonly used for patients with malignancies both in the curative and palliative settings. While it is known that radiation involving a large part of the body can indeed have impact on the bone marrow, it is rare for radiation to have a significant impact on the immune system to the point where vaccination would not be recommended. The main situation for radiation to affect immune cell generation is in the event of total body irradiation (TBI) given for marrow suppression prior to stem cell transplantation or other rare situations where patients are receiving total lymph node or spine irradiation. Therefore, most patients treated with radiation should generate protective immunity responses to COVID-19 vaccines.

## Recommendations and Conclusions

Hopes for a COVID-19 vaccine are now a reality. The BNT162b2 and mRNA-1273 mRNA vaccines are safe and highly effective (efficacy > 94%). A vaccine would be lifesaving for patients with cancer, who are at higher risk for severe COVID-19 disease and mortality than the general population. Expedited vaccination of cancer patients is therefore urgent given the continuing rise in community transmission of the disease.

Patients on cancer treatments have been excluded from COVID-19 vaccine trials thus far. Thus, we make recommendations based on what we know of the safety and efficacy of the leading vaccine candidates, performance of other vaccines in patients with cancer, and immune alterations inherent in current cancer treatments.

From a safety standpoint, trials of the leading mRNA vaccine candidates have not detected vaccine-related serious adverse events. In many cases there has been substantial reactogenicity. This is not trivial for patients with cancer, for whom (as an example) fever carries a concerning differential (e.g., infection, disease recurrence, etc.). Even so, trials find that reactogenic symptoms have generally been self-limited on the order of days. We do not anticipate safety concerns specific to patients with cancer receiving mRNA vaccines. As is true for the general public, care must be taken for those with history of anaphylaxis to components of the vaccine. Other leading candidates detailed here also appear to be generally safe. Of note, live vaccines are generally not recommended in patients undergoing targeted, cytotoxic, or lymphodepleting therapies [64–66], and a live COVID-19 vaccine should one eventually become available would also not be recommended.

From an efficacy standpoint, we expect that most cancer therapies will not inhibit the generation of protective responses by the vaccine. Lymphodepleting and intensive myelosuppressive chemotherapies will blunt the humoral and/or cell-mediated responses that are likely important for full protection against COVID-19. Nevertheless, some protection is likely beneficial. Depending on the phase and urgency of a patient's cancer treatment, there may be flexibility to optimize the timing of COVID-19 vaccinations (e.g., COVID-19 vaccination followed by anti-B cell therapy several weeks later) as is sometimes practiced for other vaccines.

In sum, we expect the leading COVID-19 vaccines to be safe and at least partly effective in patients with cancer. We recommend that patients with cancer receive COVID-19 vaccinations when they become available, acknowledging that while we do not have truly representative data, benefits likely outweigh risks. This is in keeping with the US CDC recommendation for immunocompromised individuals and the expert opinion of panelists at a joint meeting of the IDSA and ASCO following the approval of BNT162b2 [94], as well as recent guidelines and recommendations from Society of Immunotherapy of Cancer [95], the European Society for Medical Oncology [96], and the AACR COVID-19 and cancer task force [97]. Most recently, the National Comprehensive Cancer Network (NCCN) COVID-19 vaccination advisory committee have released recommendations on COVID-19 vaccines, which align with our recommendations above. Given the potentially blunted efficacy of vaccines, as well as the lack of data thus far on preventing asymptomatic transmission or durability of protection, we recommend that caregivers of patients with cancer be vaccinated and that all vaccine recipients continue social distancing, hygiene, and mask precautions. We further advocate for the creation of a national registry for patients with cancer receiving active cancer treatment and COVID-19 vaccines to facilitate clinical trials of patients with cancer receiving these vaccines, which will be the key to fully elucidate the safety, immunogenicity, and efficacy of COVID-19 vaccines in this complex population. Collectively, we will see our patients safely through this pandemic.

## Data Availability

The material supporting the conclusion of this review has been included within the article.

## Abbreviations

ADE: Antibody-dependent enhancement
aOR: Adjusted odds ratio
CD20: Cluster of differentiation 20
CD38: Cluster of differentiation 38
CD4: Cluster of differentiation 4
CD8: Cluster of differentiation 8
COVID-19: Coronavirus disease 2019
CTLA-4: Cytotoxic T-lymphocyte-associated protein 4
DNA: Deoxyribonucleic acid
ECIL: European Conference on Infections in Leukemia
FDA: US Food and Drug Administration
HIV: Human immunodeficiency virus
IDSA: Infectious Diseases Society of America
IgG: Immunoglobulin G
IRAE: Immune-related adverse events
LNP: Lipid nanoparticle
MHC: Major histocompatibility complex
mRNA: Messenger RNA
NCCN: National Comprehensive Cancer Network
PD-1: Programmed cell death protein 1
RNA: Ribonucleic acid
SARS-CoV-2: Severe acute respiratory syndrome coronavirus 2
Th1: T-helper lymphocyte type 1
Th2: T-helper lymphocyte type 2
TKI: Tyrosine kinase inhibitors

## References

[1] Deng W, Bao L, Liu J, Xiao C, Liu J, Xue J (2020). Primary exposure to SARS-CoV-2 protects against reinfection in rhesus macaques. Science.

[2] Wajnberg A, Amanat F, Firpo A, Altman DR, Bailey MJ, Mansour M (2020). Robust neutralizing antibodies to SARS-CoV-2 infection persist for months. Science..

[3] Rogers TF, Zhao F, Huang D, Beutler N, Burns A, He W-T (2020). Isolation of potent SARS-CoV-2 neutralizing antibodies and protection from disease in a small animal model. Science.

[4] Hassan AO, Case JB, Winkler ES, Thackray LB, Kafai NM, Bailey AL (2020). A SARS-CoV-2 infection model in mice demonstrates protection by neutralizing antibodies. Cell.

[5] Libster R, Pérez Marc G, Wappner D, Coviello S, Bianchi A, Braem V, et al. Early High-Titer Plasma Therapy to Prevent Severe Covid-19 in Older Adults. N Engl J Med. 2021;NEJMoa2033700. 10.1056/NEJMoa2033700 PMC7793608 33406353

[6] Garcia-Beltran WF, Lam EC, Astudillo MG, Yang D, Miller TE, Feldman J, et al. COVID-19-neutralizing antibodies predict disease severity and survival. Cell. 2021;184(2):476–88 e11. 10.1016/j.cell.2020.12.015 PMC7837114 33412089

[7] Ni L, Ye F, Cheng M-L, Feng Y, Deng Y-Q, Zhao H (2020). Detection of SARS-CoV-2-specific humoral and cellular immunity in covid-19 convalescent individuals. Immunity.

[8] Grifoni A, Weiskopf D, Ramirez SI, Mateus J, Dan JM, Moderbacher CR (2020). Targets of T cell responses to SARS-CoV-2 coronavirus in humans with COVID-19 disease and unexposed individuals. Cell.

[9] Chen Y, Zuiani A, Fischinger S, Mullur J, Atyeo C, Travers M (2020). Quick COVID-19 healers sustain anti-SARS-CoV-2 antibody production. Cell.

[10] Dan JM, Mateus J, Kato Y, Hastie KM, Yu ED, Faliti CE (2021). Immunological memory to SARS-CoV-2 assessed for up to eight months after infection. Science..

[11] Rodda LB, Netland J, Shehata L, Pruner KB, Morawski PA, Thouvenel CD (2021). Functional SARS-CoV-2-Specific Immune Memory Persists after Mild COVID-19. CCell..

[12] Bolles M, Deming D, Long K, Agnihothram S, Whitmore A, Ferris M (2011). A double-inactivated severe acute respiratory syndrome coronavirus vaccine provides incomplete protection in mice and induces increased eosinophilic proinflammatory pulmonary response upon challenge. J Virol.

[13] Tseng C-T, Sbrana E, Iwata-Yoshikawa N, Newman PC, Garron T, Atmar RL (2012). Immunization with SARS coronavirus vaccines leads to pulmonary immunopathology on challenge with the SARS virus. PLoS ONE.

[14] Gao Q, Bao L, Mao H, Wang L, Xu K, Yang M (2020). Development of an inactivated vaccine candidate for SARS-CoV-2. Science.

[15] Wang H, Zhang Y, Huang B, Deng W, Quan Y, Wang W (2020). Development of an inactivated vaccine candidate, BBIBP-CorV, with potent protection against SARS-CoV-2. Cell.

[16] Lee WS, Wheatley AK, Kent SJ, DeKosky BJ (2020). Antibody-dependent enhancement and SARS-CoV-2 vaccines and therapies. Nat Microbiol.

[17] Simonovich VA, Burgos Pratx LD, Scibona P, Beruto MV, Vallone MG, Vázquez C, et al. A Randomized Trial of Convalescent Plasma in Covid-19 Severe Pneumonia. N Engl J Med. 2020:NEJMoa2031304. 10.1056/NEJMoa2031304 PMC7722692 33232588

[18] Agarwal A, Mukherjee A, Kumar G, Chatterjee P, Bhatnagar T, Malhotra P (2020). Convalescent plasma in the management of moderate covid-19 in adults in India: open label phase II multicentre randomised controlled trial (PLACID Trial). BMJ.

[19] Krammer F (2020). SARS-CoV-2 vaccines in development. Nature.

[20] Haq EU, Yu J, Guo J (2020). Frontiers in the COVID-19 vaccines development. Exp Hematol Oncol.

[21] Zhang Y, Zeng G, Pan H, Li C, Hu Y, Chu K (2021). Safety, tolerability, and immunogenicity of an inactivated SARS-CoV-2 vaccine in healthy adults aged 18–59 years: a randomised, double-blind, placebo-controlled, phase 1/2 clinical trial. Lancet Infect Dis..

[22] Xia S, Zhang Y, Wang Y, Wang H, Yang Y, Gao GF (2021). Articles Safety and immunogenicity of an inactivated SARS-CoV-2 vaccine, BBIBP-CorV: a randomised, double-blind, placebo-controlled, phase 1/2 trial. Lancet Infect Dis..

[23] Keech C, Albert G, Cho I, Robertson A, Reed P, Neal S (2020). Phase 1–2 trial of a SARS-CoV-2 recombinant spike protein nanoparticle vaccine. N Engl J Med.

[24] Voysey M, Clemens SAC, Madhi S, Weckx L, Folegatti PM, Aley PK, et al. Safety and efficacy of the ChAdOx1 nCoV-19 vaccine (AZD1222) against SARS-CoV-2: an interim analysis of four randomised controlled trials in Brazil, South Africa, and the UK. The Lancet. 20221;397(10269):99–111. 10.1016/S0140-6736(20)32661-1 PMC7723445 33306989

[25] Folegatti PM, Ewer KJ, Aley PK, Angus B, Becker S, Belij-Rammerstorfer S, et al. Safety and immunogenicity of the ChAdOx1 nCoV-19 vaccine against SARS-CoV-2: a preliminary report of a phase 1/2, single-blind, randomised controlled trial. The Lancet. 2020;396(10249):467–78. 10.1016/S0140-6736(20)31604-4 PMC7445431 32702298

[26] Wu S, Zhong G, Zhang J, Shuai L, Zhang Z, Wen Z (2020). A single dose of an adenovirus-vectored vaccine provides protection against SARS-CoV-2 challenge. Nat Commun.

[27] Zhu F-C, Guan X-H, Li Y-H, Huang J-Y, Jiang T, Hou L-H (2020). Immunogenicity and safety of a recombinant adenovirus type-5-vectored COVID-19 vaccine in healthy adults aged 18 years or older: a randomised, double-blind, placebo- controlled, phase 2 trial. The Lancet.

[28] Mercado NB, Zahn R, Wegmann F, Loos C, chandrashekar a, Yu J, et al. Single-shot Ad26 vaccine protects against SARS-CoV-2 in rhesus macaques. Nature. 2020:1–22. 10.1038/s41586-020-2607-z PMC7581548 32731257

[29] Sadoff J, Le Gars M, Shukarev G, Heerwegh D, Truyers C, de Groot AM, et al. Interim Results of a Phase 1-2a Trial of Ad26.COV2.S Covid-19 Vaccine. N Engl J Med. 2021; NEJMa2034201. 10.1056/NEJMoa2034201 PMC7821985 33440088

[30] Logunov DY, Dolzhikova IV, Zubkova OV, Tukhvatullin AI, Shcheblyakov DV, Dzharullaeva AS (2020). Safety and immunogenicity of an rAd26 and rAd5 vector-based heterologous prime-boost COVID-19 vaccine in two formulations: two open, non-randomised phase 1/2 studies from Russia. Lancet.

[31] CDC. Emerging SARS-CoV-2 Variants 2021 [Available from: https://www.cdc.gov/coronavirus/2019-ncov/more/science-and-research/scientific-brief-emerging-variants.html.

[32] Yu J, Tostanoski LH, Peter L, Mercado NB, McMahan K, Mahrokhian SH (2020). DNA vaccine protection against SARS-CoV-2 in rhesus macaques. Science.

[33] Liu MA (2019). A comparison of plasmid DNA and mRNA as vaccine technologies. Vaccines.

[34] Pardi N, Hogan MJ, Porter FW, Weissman D (2018). mRNA vaccines—a new era in vaccinology. Nature Publishing Group.

[35] Cafri G, Gartner JJ, Zaks T, Hopson K, Levin N, Paria BC (2020). mRNA vaccine-induced neoantigen-specific T cell immunity in patients with gastrointestinal cancer. J Clin Investig.

[36] Fiedler K, Lazzaro S, Lutz J, Rauch S, Heidenreich R (2016). mRNA cancer vaccines. Recent results in cancer research Fortschritte der Krebsforschung Progres dans les recherches sur le cancer.

[37] Mitchell MJ, Billingsley MM, Haley RM, Wechsler ME, Peppas NA, Langer R (2020). Engineering precision nanoparticles for drug delivery. Nat Publ Group.

[38] Şahin U, Karikó K, Türeci Ö (2014). mRNA-based therapeutics–developing a new class of drugs. Nat Publ Group.

[39] Feldman RA, Fuhr R, Smolenov I, Ribeiro AM, Panther L, Watson M (2019). mRNA vaccines against H10N8 and H7N9 influenza viruses of pandemic potential are immunogenic and well tolerated in healthy adults in phase 1 randomized clinical trials. Vaccine.

[40] Walsh EE, Frenck RW, Falsey AR, Kitchin N, Absalon J, Gurtman A (2020). Safety and immunogenicity of two RNA-based Covid-19 vaccine candidates. N Engl J Med.

[41] Sahin U, Muik A, Vogler I, Derhovanessian E, Kranz LM, Vormehr M, et al. BNT162b2 induces SARS-CoV-2-neutralising antibodies and T cells in humans. medRxiv. 2020.

[42] Polack FP, Thomas SJ, Kitchin N, Absalon J, Gurtman A, Lockhart S (2020). Safety and Efficacy of the BNT162b2 mRNA Covid-19 Vaccine. N Engl J Med.

[43] Sahin U, Muik A, Vogler I, Derhovanessian E, Vogler I, Kranz LM, Vormehr M (2020). COVID-19 vaccine BNT162b2 elicits human antibody and TH1 T-cell responses. Nature..

[44] Widge AT, Rouphael NG, Jackson LA, Anderson EJ, Roberts PC, Makhene M, et al. Durability of Responses after SARS-CoV-2 mRNA-1273 Vaccination. N Engl J Med. 2021;384(1):80–2. 10.1056/NEJMc2032195 PMC7727324 33270381

[45] Baden LR, El Sahly HM, Essink B, Kotloff K, Frey S, Novak R (2021). Efficacy and Safety of the mRNA-1273 SARS-CoV-2 Vaccine. N Engl J Med..

[46] Oliver SE, Gargano JW, Marin M, Wallace M, Curran KG, Chamberland M, et al. The Advisory Committee on Immunization Practices’ Interim Recommendation for Use of Moderna COVID-19 Vaccine — United States, December 2020. 2020:1–4. 10.15585/mmwr.mm695152e1 PMC9191904 33382675

[47] Administration USFaD. Vaccines and Related Biological Products Advisory Committee Meeting December 17, 2020. 2020 [Available from: https://www.fda.gov/media/144434/download.

[48] Williamson EJ, Walker AJ, Bhaskaran K, Bacon S, Bates C, Morton CE, et al. Factors associated with COVID-19-related death using OpenSAFELY. Nature. 2020:1–17. 10.1038/s41586-020-2521-4 PMC7611074 32640463

[49] Rivera DR, Peters S, Panagiotou OA, Shah DP, Kuderer NM, Hsu C-Y (2020). Utilization of COVID-19 Treatments and Clinical Outcomes among Patients with Cancer: A COVID-19 and Cancer Consortium (CCC19) Cohort Study. Cancer Discov.

[50] Kuderer NM, MD TKC, PhD DPS, PhD YS, MD SMR, PharmD DRR, et al. Clinical impact of COVID-19 on patients with cancer (CCC19): a cohort study. The Lancet. 2020;395(10241):1907–18. 10.1016/S0140-6736(20)31187-9 PMC7255743 32473681

[51] Garcia-Suarez J, de la Cruz J, Cedillo A, Llamas P, Duarte R, Jimenez-Yuste V (2020). Impact of hematologic malignancy and type of cancer therapy on COVID-19 severity and mortality: lessons from a large population-based registry study. J Hematol Oncol.

[52] Wang Q, Berger NA, Xu R. Analyses of risk, racial disparity, and outcomes among US patients with cancer and COVID-19 infection. JAMA Oncol. 2020:1–8. 10.1001/jamaoncol.2020.6178 PMC7729584 33300956

[53] Ménétrier-Caux C, Ray-Coquard I, Blay J-Y, Caux C. Lymphopenia in cancer patients and its effects on response to immunotherapy: an opportunity for combination with cytokines? 2019:1–15. 10.1186/s40425-019-0549-5 PMC6437964 30922400

[54] Yu JW, Borkowski A, Danzig L, Reiter S, Kavan P, Mazer BD (2007). Immune response to conjugated meningococcal C vaccine in pediatric oncology patients. Pediatr Blood Cancer.

[55] Goyal S, Pai SK, Kelkar R, Advani SH (1998). Hepatitis B vaccination in acute lymphoblastic leukemia. Leuk Res.

[56] Ercan TE, Soycan LY, Apak H, Celkan T, Ozkan A, Akdenizli E (2005). Antibody titers and immune response to diphtheria-tetanus-pertussis and measles-mumps-rubella vaccination in children treated for acute lymphoblastic leukemia. J Pediatr Hematol Oncol.

[57] Lo W, Whimbey E, Elting L, Couch R, Cabanillas F, Bodey G (1993). Antibody response to a two-dose influenza vaccine regimen in adult lymphoma patients on chemotherapy. Eur J Clin Microbiol Infect Dis Offic Publ Eur Soc Clin Microbiol.

[58] Mazza JJ, Yale SH, Arrowood JR, Reynolds CE, Glurich I, Chyou P-H (2005). Efficacy of the influenza vaccine in patients with malignant lymphoma. Clin Med Res.

[59] Nordøy T, Aaberge IS, Husebekk A, Samdal HH, Steinert S, Melby H, et al. Cancer Patients Undergoing chemotherapy show adequate serological response to vaccinations against influenza virus and streptococcus pneumoniae. Med Oncol. 2002:19(2):71–8 10.1385/MO:19:2:71 12180483

[60] Wumkes ML, van der Velden AMT, Los M, Leys MBL, Beeker A, Nijziel MR (2013). Serum antibody response to influenza virus vaccination during chemotherapy treatment in adult patients with solid tumours. Vaccine.

[61] Anderson H, Petrie K, Berrisford C, Charlett A, Thatcher N, Zambon M. Seroconversion after influenza vaccination in patients with lung cancer. 1999:1–2. 10.1038/sj.bjc.6690342 PMC2362985 10389999

[62] Meerveld-Eggink A, de Weerdt O, van der Velden AMT, Los M, van der Velden AWG, Stouthard JML (2011). Response to influenza virus vaccination during chemotherapy in patients with breast cancer. Ann Oncol Offic J Eur Soc Med Oncol.

[63] Keam B, Kim M-K, Choi Y, Choi S-J, Choe PG, Lee K-H (2016). Optimal timing of influenza vaccination during 3-week cytotoxic chemotherapy cycles. Cancer.

[64] Rubin LG, Levin MJ, Ljungman P, Davies EG, Avery R, Tomblyn M (2013). 2013 IDSA clinical practice guideline for vaccination of the immunocompromised host. Clin Infect Dis.

[65] Mikulska M, Cesaro S, de Lavallade H, Di Blasi R, Einarsdottir S, Gallo G, et al. Vaccination of patients with haematological malignancies who did not have transplantations: guidelines from the 2017 European Conference on Infections in Leukaemia (ECIL 7). The Lancet Infectious Diseases. 2019;19(6):e188-e99. 10.1016/S1473-3099(18)30601-7 30744964

[66] Rieger CT, Liss B, Mellinghoff S, Buchheidt D, Cornely OA, Egerer G (2018). Anti-infective vaccination strategies in patients with hematologic malignancies or solid tumors—Guideline of the Infectious Diseases Working Party (AGIHO) of the German Society for Hematology and Medical Oncology (DGHO). Ann Oncol.

[67] Kersh AE, Ng S, Chang YM, Sasaki M, Thomas SN, Kissick HT (2017). Targeted therapies: immunologic effects and potential applications outside of cancer. J Clin Pharmacol.

[68] de Lavallade H, Khoder A, Hart M, Sarvaria A, Sekine T, Alsuliman A (2013). Tyrosine kinase inhibitors impair B-cell immune responses in CML through off-target inhibition of kinases important for cell signaling. Blood.

[69] Mulder SF, Jacobs JFM, Olde Nordkamp MAM, Galama JMD, Desar IME, Torensma R (2011). Cancer patients treated with sunitinib or sorafenib have sufficient antibody and cellular immune responses to warrant influenza vaccination. Clin Cancer Res Offic J Am Assoc Cancer Res.

[70] Joona TB, Digkas E, Wennstig A-K, Nyström K, Nearchou A, Nilsson C (2020). Influenza vaccination in breast cancer patients during subcutaneous trastuzumab in adjuvant setting. Breast Cancer Res Treat.

[71] Sun C, Gao J, Couzens L, Tian X, Farooqui MZ, Eichelberger MC (2016). Seasonal influenza vaccination in patients with chronic lymphocytic leukemia treated with ibrutinib. JAMA Oncol.

[72] Douglas AP, Trubiano JA, Barr I, Leung V, Slavin MA, Tam CS (2017). Ibrutinib may impair serological responses to influenza vaccination. Haematologica..

[73] Zent CS, Brady MT, Delage C, Strawderman M, Laniewski N, Contant PN, et al. Short term results of vaccination with adjuvanted recombinant varicella zoster glycoprotein E during initial BTK inhibitor therapy for CLL or lymphoplasmacytic lymphoma. Leukemia. 2020:1–4. 10.1038/s41375-020-01074-4 PMC7596619 33128020

[74] Mehta V, Goel S, Kabarriti R, Cole D, Goldfinger M, Acuna-Villaorduna A (2020). Case fatality rate of cancer patients with COVID-19 in a New York Hospital System. Cancer Discov.

[75] Garassino MC, Whisenant JG, Huang LC, Trama A, Torri V, Agustoni F (2020). COVID-19 in patients with thoracic malignancies (TERAVOLT): first results of an international, registry-based, cohort study. Lancet Oncol.

[76] Luo J, Rizvi H, Egger JV, Preeshagul IR, Wolchok JD, Hellmann MD (2020). Impact of PD-1 blockade on severity of COVID-19 in patients with lung cancers. Cancer Discov.

[77] Wang P-F, Chen Y, Song S-Y, Wang T-J, Ji W-J, Li S-W (2017). Immune-related adverse events associated with Anti-PD-1/PD-L1 treatment for malignancies: a meta-analysis. Frontiers Pharmacol.

[78] Läubli H, Balmelli C, Kaufmann L, Stanczak M, Syedbasha M, Vogt D (2018). Influenza vaccination of cancer patients during PD-1 blockade induces serological protection but may raise the risk for immune-related adverse events. J Immunother Cancer.

[79] Gambichler T, Reuther J, Scheel CH, Becker JC (2020). On the use of immune checkpoint inhibitors in patients with viral infections including COVID-19. J Immunother Cancer.

[80] Keam B, Kang CK, Jun KI, Moon SM, Suh KJ, Lee D-W (2020). Immunogenicity of influenza vaccination in patients with cancer receiving immune checkpoint inhibitors. Clin Infect Dis.

[81] Chen G, Wu Q, Jiang H, Li Z, Hua X, Hu X (2020). Impact of treatment delay due to the pandemic of COVID-19 on the efficacy of immunotherapy in head and neck cancer patients. J Hematol Oncol.

[82] Kneitz C, Wilhelm M, Tony HP (2002). Effective B cell depletion with rituximab in the treatment of autoimmune diseases. Immunobiology.

[83] Cho A, Bradley B, Kauffman R, Priyamvada L, Kovalenkov Y, Feldman R (2017). Robust memory responses against influenza vaccination in pemphigus patients previously treated with rituximab. JCI Insight.

[84] Nazi I, Kelton JG, Larché M, Snider DP, Heddle NM, Crowther MA (2013). The effect of rituximab on vaccine responses in patients with immune thrombocytopenia. Blood.

[85] Berglund Å, Willén L, Grödeberg L, Skattum L, Hagberg H, Pauksens K (2014). The response to vaccination against influenza A(H1N1) 2009, seasonal influenza and Streptococcus pneumoniae in adult outpatients with ongoing treatment for cancer with and without rituximab. Acta oncologica (Stockholm, Sweden).

[86] Bouaziz J-D, Yanaba K, Venturi GM, Wang Y, Tisch RM, Poe JC (2007). Therapeutic B cell depletion impairs adaptive and autoreactive CD4. Proceed Nat Acad Sci..

[87] Preliminary recommendations of the NCCN COVID-19 Vaccination Advisory Committee [press release]. 2021.

[88] Bhoj VG, Arhontoulis D, Wertheim G, Capobianchi J, Callahan CA, Ellebrecht CT (2016). Persistence of long-lived plasma cells and humoral immunity in individuals responding to CD19-directed CAR T-cell therapy. Blood.

[89] Krejcik J, Casneuf T, Nijhof IS, Verbist B, Bald J, Plesner T (2016). Daratumumab depletes CD38+ immune regulatory cells, promotes T-cell expansion, and skews T-cell repertoire in multiple myeloma. Blood.

[90] Frerichs KA, Bosman PW, van Velzen JF, Fraaij PLA, Koopmans MPG, Rimmelzwaan GF (2020). Effect of daratumumab on normal plasma cells, polyclonal immunoglobulin levels, and vaccination responses in extensively pre-treated multiple myeloma patients. Haematologica..

[91] Hill JA, Seo SK (2020). How I prevent infections in patients receiving CD19-targeted chimeric antigen receptor T cells for B-cell malignancies. Blood.

[92] Loarce-Martos J, García-Fernández A, López-Gutiérrez F, García-García V, Calvo-Sanz L, Del Bosque-Granero I (2020). High rates of severe disease and death due to SARS-CoV-2 infection in rheumatic disease patients treated with rituximab: a descriptive study. Rheumatol Int.

[93] Damiani G, Pacifico A, Bragazzi NL, Malagoli P. Biologics increase the risk of SARS‐ CoV‐2 infection and hospitalization, but not ICUadmission and death: Real‐life data from a large cohort during red‐zonedeclaration. Dermatologic Therapy. 2020;33(5):CD011972–6. 10.1111/dth.13475 PMC7261990 32356577

[94] Oncology ASoC. COVID-19 Vaccine & Patients with Cancer. 2020. Available from: https://www.asco.org/asco-coronavirusresources/covid-19-patient-care-information/covid-19-vaccine-patients-cancer.

[95] Cancer SfIo. SITC Statement on SARS-CoV-2 Vaccination and Cancer Immunotherapy. 2020. Available from: https://www.sitcancer.org/aboutsitc/press-releases/2020/sitc-statement-sars-cov-2-vaccination-cancer-immunotherapy.

[96] Oncology ESfM. ESMO Statements for vaccination against COVID-19 in patients with cancer. 2020. Available from: https://www.esmo.org/covid-19-and-cancer/covid-19-vaccination.

[97] Ribas A, Sengupta R, Locke T, Zaidi SK, Campbell KM, Carethers JM (2021). Priority COVID-19 vaccination for patients with cancer while vaccine supply is limited. Cancer Discov..

